# Neighborhood-level heterogeneity in childhood morbidity through generalized linear mixed models

**DOI:** 10.3389/fpubh.2025.1456068

**Published:** 2025-07-21

**Authors:** Endeshaw A. Derso, Kassahun A. Gelaye, Maria G. Campolo, Amare T. Woldemariam, Angela Alibrandi

**Affiliations:** ^1^Department of Economics, University of Messina, Messina, Italy; ^2^Department of Statistics, College of Natural and Computational Science, University of Gondar, Gondar, Ethiopia; ^3^Lihiket (Excellence) Institutional Development and Postdoctoral Fellowship Programme, Department of Epidemiology and Biostatistics, Institute of Public Health, University of Gondar, Gondar, Ethiopia; ^4^Department of Human Nutrition, Institute of Public Health, College of Medicine and Health Sciences, University of Gondar, Gondar, Ethiopia; ^5^Department of Women's and Children's Health, Karoliniska Institutet, Stockholm, Sweden

**Keywords:** AIC, children comorbidity, DHARMa, GLMMs, Laplace approximation, random effect

## Abstract

**Objective:**

Childhood morbidities are crucial for improving long-term public health outcomes. This study aimed to examine the existence of child-specific and regional variation in childhood morbidity based on the cross-cutting study of the Performance Monitoring for Action Ethiopia community survey (PMA-ET), and its relationship to socioeconomic and demographic variables in families.

**Methods:**

We enrolled 2,581 children suffering from different illnesses from six regions of the country of the survey at 6 weeks postpartum. Generalized linear mixed models (GLMMs) with maximum likelihood estimation were used to assess children's comorbidity status, and the DHARMa package in R to provide readily interpretable scaled residuals and test functions for typical model misspecification problems for the fitted GLMMs.

**Results:**

GLMMs with two random intercept models show the presence of child morbidity variations. Cough, fever, and diarrhea were found to be the most frequent types of children's illnesses among the main illness categories that were recorded. Cooking fuel, wealth quartiles, mothers' marital status, mother age, parity, residence, mother's education status, and availability of electricity were significantly associated with children's morbidity.

**Conclusions:**

These data show that variations in children's comorbidity were associated with both regional and child-specific characteristics. Thus, general principles for designing policies and interventions are required to reduce child comorbidity.

## 1 Introduction

Child morbidity the perception of being unwell as a result of specific conditions or illnesses. This term pertains to the prevalence of health issues that affect the wellbeing of children. Infectious diseases, such as pneumonia, diarrhea, and malaria, are the leading causes of global under-five child deaths (1, 2). Alarming statistics reveal that two-thirds of global child mortality occurs in underdeveloped countries. As a result, in order to reduce global child mortality, the urgent need to address child morbidity in low and middle income countries is evident, particularly as these region are struggling to meet the Sustainable Development Goal (SDG) target related to child mortality reduction (target 3.2), which aims to bring child deaths per thousand live birth down to 25 by the year 2023 (3, 4).

While there has been a notable decline in global under-five child mortality in recent decades, the progress in developing and under-developed regions, such as Africa and Bangladesh, has not been satisfactory (4, 5). Reports from the World Health Organization indicate that the Sub-Saharan Africa and the South Asia bear the burden of ~80% of all child deaths worldwide (6). A recent study conducted in Tanzania stated that, 63 child deaths per thousand live births occurred in the 2016 estimate in Tanzania, though it was declined by 42%. A separated study conducted in Tanzanian, drawing data from 35 hospitals, identified respiratory distress as the primary cause of early neonatal death, accounting for approximately 21% of cases (4, 7, 8). Despite the fact that Ethiopia's infant mortality rate fell from 34.010 deaths per 1,000 live births in 2020 to 29.524 deaths per 1,000 live births in 2023 (9), child morbidity was still significant, particularly among children under the age of one (10).

Therefore, to successfully design a national program for childhood morbidity intervention, it is necessary to identify determinants in a local context. Hence, several earlier studies suggested that environmental, socioeconomic, demographic, and health-associated factors lead to childhood morbidity globally (11–18). For instance, mother's age, mother's education, family wealth, handwashing, sanitation, child's gender, child's anemia level, husband's education level, mother's job status, mother's marital status, breastfeeding status, and exposure to morbidity information have been found to have an effect on child morbidity (10, 12, 13, 15, 19–28). Two-parent families have more stable family structures and stronger social support networks for their children's to improve their child health (29–31). Likewise, the rate of children's illness also differs across geographical regions, their residence, high-parity-births, and the availability of electricity (11, 15, 32–35). Obstructive sleep apnea (OSA) can also cause serious morbidity in middle-age women and even it may lead to an increased risk of high blood pressure, high cholesterol, prediabetes, and other heart and blood vessel conditions in children (36, 37), compared to older children, infants with OSA have different comorbidities (38).

Furthermore, previous studies in Ethiopia have identified a wide range of risk factors, including socioeconomic, environmental, demographic, and other elements that influence childhood morbidity (35, 39–43). However, most of these studies focus on predicting factors associated with a single health condition, even though children in Ethiopia suffer from multiple health problems due to limited access to health services and poor household socioeconomic environments in the country. Furthermore, understanding the cause and expected outcome of morbidity in children will be insufficient if the focus is on specific diseases or categories of illnesses (44, 45). Besides, previous studies also did not account for potential variation among clusters of individuals or groups and none of them deals with insights to unravel the intricate relationship between child health and cluster context in Ethiopia. Thus, to account for this source of variability, we propose a generalized linear mixed models (GLMMs) that can be used to analyze data that is collected from multiple subjects within different clusters or clustered data, and handle random effects that used to model the variability in the response variable due to the grouping structure of the data (46). GLMMs can model both common and individual behaviors, contain more information, have more variability, and are more efficient than pure time series or cross-sectional data (47).

Therefore, in this paper, we specifically focus on studying within-subject variation and between-subject effects in GLMMs to understand how child comorbidity varies within and between subjects by considering the child's id and region as random effects and to identify the factors associated with this heterogeneity. Our model incorporates diverse potential predictors for comorbidity sourced from the 2019 Performance Monitoring for Action Ethiopia (PMA-ET) community survey datasets. These datasets systematically gather information on child health and household characteristics, drawing from a nationally representative sample of households. It's worth noting that this dataset captures valuable information that is presently underutilized by other extensive surveys, such as demographic and health surveys like DHS (48).

In terms of parameter estimation, a likelihood-based approach is often recommended, with Akaike's information criteria serving as a tool for model selection in likelihood-based estimation (49). Furthermore, we use a simulation-based approach of the DHARMa package in R to create readily interpretable scaled (quintile) residuals for fitted GLMMs (50). Our analysis of advanced current methodological approaches with a recent data set of interest will provide robust information for the best possible planning of health services as well as a better understanding of the state of children's health.

## 2 Materials and methods

PMA Ethiopia generates timely cross-sectional and longitudinal data on reproductive, maternal, and new-born health indicators. We use the data from a nationally representative longitudinal study (cohort one study) conducted from October 2019 to August 2021 which collects details on mothers' characteristics and child health from a nationally representative sample of households.

### 2.1 Sampling and study design settings

PMA Ethiopia used a sampling method called multistage stratified cluster sampling to select households for their study. They selected households from specific clusters or enumeration areas (EAs), with the areas being chosen based on their size within different groups. In some regions, the strata were determined by the region and whether it was urban or rural, while in other regions, the strata were just based on the regions themselves. Within the regions that were part of the study, a census was conducted to identify all households and women between the ages of 15–49 who were regular members of the household.

**Figure 1:**
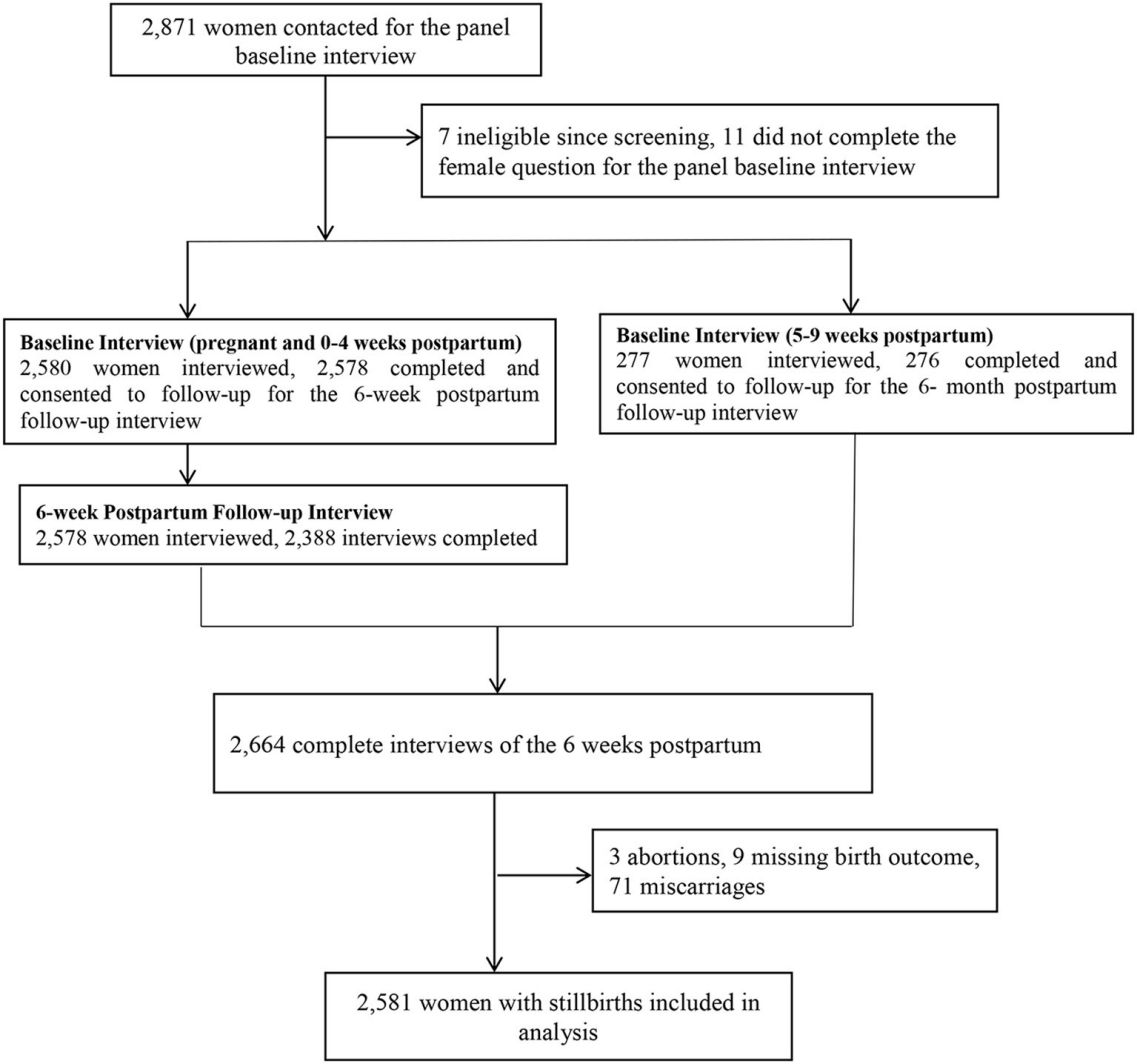
Data extraction for 6 week postpartum follow-up interview flowchart.

All women who were aged 15–49 were screened, and those who reported being pregnant or having given birth in the past 6 weeks were eligible for the survey. Explicit inclusion criteria consisted of women aged 15–49 years who had recent births, as defined by the PMA-ET survey design. No additional exclusion criteria were applied due to limitations inherent in the original dataset, which did not provide further exclusion-related information. From this group, consenting eligible women were enrolled in the study and completed a baseline interview and were then reinter viewed at 6 weeks, 6 months, and 1 year postpartum by a trained interviewer. This study employs a cross-sectional analysis using data collected from baseline and six-week postpartum interviews. Specifically, we analyzed child morbidity variation and its association with socio-demographic, maternal, and child health-related factors at the six-week postpartum period. The baseline interview collected information about women's socio-demographic characteristics. PMA-ET was able to interview the minimum number of women per EA and achieve a sample that was representative on both national and regional levels. During the interview, women were asked about the socioeconomic characteristics of their households and the health status of their children. Among the 2,871 women contacted for the panel baseline interview, 2,855 enrolled pregnant or recently post-partum women in our survey, 2,664 completed six-week postpartum interviews (conducted at baseline and 6 weeks postpartum), 2,581 women with live births used for the analysis (71 women with miscarriage or abortion excluded; see Figure 1). Confounding variables were selected based on consistent associations reported in previous studies on child morbidity in similar settings; the validity and reliability of the data collection instruments were established by the original PMA-ET study team (48).

### 2.2 The variables

Our study includes a range of potential predictors for child comorbidity from the PMA-ET dataset (see Table 1), including the mother's age, mother's education, mother's parity, region, residence, types of cooking fuel, sanitary classification, availability of electricity, and wealth. The outcome variable considered is binary, taking a value of one if a child developed at least one complication (namely cough, fever, diarrheal, vomiting, eye infection, skin rash, poor feeding, difficulty breathing, etc.) in the postpartum interview.

**Table 1:** Sociodemographic covariates and their labeling for child comorbidity study.

**Variables**	**Variable description and/or classification and labeling**
Cooking fuel	Electricity =1, kerosene = 2, charcoal = 3, and wood = 4
Wealth	Household wealth quantiles: lower quartiles = 1, middle quartiles =2 and higher quartiles = 3
Sanitation classification	Improved, not shared facility =1, shared facility = 2, non-improved facility =3, and Open defecation = 4
Residence	Urban =1, and rural = 2
Education	Never attended = 0, primary education =1, secondary education= 2, above secondary education =3
Marital	Married or with partner = 1, widowed or divorced = 2, and never married =3
Age	Age between 15–24 = 1, Age between 25–34 = 2 and Age above 34 = 3
Parity	Zero parity = 0, parity between 1 and 2 =1, parity between 3 and 4 = 2, and parity above 4 = 3
Electricity availability	No = 1 and yes = 2
Region	Tigray, Afar, Amhara, Oromia, SNNP, and Addis Ababa

Notational


$$y=\{\begin{array}{cc} 1, & if\;the\;child\;suffers\;from\;at\;least\;one\;of\;major\;complication\\ 0, & otherwise \end{array}$$

Considering the random effects data utilized in this study, we used the child's id to visualize an interclass correlation while the six regions represent the intraclass correlation to capture the variation in child comorbidity of our study. Thus, samples were grouped by six different regions of the country, namely Afar, Amhara, Oromia, Tigray SNNP, and Addis Ababa. Analyzing categorical variables in GLMMs, one of the categories is used as a reference category, and the other categories are then measured against the reference category in analyzing categorical variables in GLMMs (46). Besides, region and child's ID are uniquely labeled; we can specify random effects as (1|region) and (1|child_ID).

The categorization of sociodemographic variables (Table 1) was based on commonly used groupings in existing literature related to child morbidity and public health research in similar settings (11, 13). Age groups, education levels, wealth indices, and residence status were categorized following standard demographic health survey (DHS) practices to maintain comparability across studies. Missing data were assessed prior to analysis; the PMA-ET dataset underwent a rigorous quality control process, and minimal missingness was detected. During our data cleaning phase, we verified the completeness of key variables using frequency distributions and summary statistics, confirming that there were no significant missing values in the final analytic sample. Sensitivity analysis was not performed, as the minimal level of missing data and the robust sample size were deemed sufficient to support the stability of the primary findings.

### 2.3 Methods

#### 2.3.1 Generalized linear mixed model

Generalized Linear Mixed Models (GLMMs) were developed to address the need for analyzing non-normally distributed responses that exhibit correlation or clustering. GLMMs account for variation in cluster data by incorporating random effects into the model to capture the heterogeneity of observation within clusters (51–53). GLMMs are used for fully parametric subject-specific inference for clustered or repeated measurement responses in the exponential family (54). It is particularly useful in biomedical studies as it can account for the correlation between observations that arise from the hierarchical structure of the data and in recent years, the use of GLMMs in Biomedical study has increased, and now it is considered one of the most powerful and challenging tools in the field (55, 56). The link function had been used to account for the correlation between the data within each cluster and to model non-normal outcome variables. In GLMMs, the logit link function maximizes the likelihood of the data under the model, maps the probability of a binary outcome to a linear predictor, and has straightforward interpretations in terms of the odds ratio (53, 57, 58).

#### 2.3.2 Model specification of GLMMs

Let *y*_*ij*_ be the binary response measured for *i*^*th*^ cluster, for *i* = *1, 2,…,N, j* = *1,2,…*, *n*_*i*_, and *x*_*ij*_ is the *i*th row of the matrix for the fixed effects and *y*_*i*_ is the *n*_*i*_-dimension vectors of all measurements available for *i*^*th*^ child, conditional on the random vector *b*_*i*_ with *q* dimensions, and which is assumed to be drawn independently from a distribution belong to exponential family. Furthermore, *b*_*i*_ captures the unobserved factors specific to each cluster that affect the child comorbidity and is assumed to be drawn independently from the normal distribution with mean zero and variance ${\sigma_{b}}^{2}$ i.e. *b*_*i*_ ~*N(0*, ${\sigma_{b}}^{2}$*)*, where ${\sigma_{b}}^{2}$ refers to the variation in the population distribution and, consequently, the degree of subject heterogeneity. Thus, the probability density function of the response *y*_*ij*_, which is independent of the distribution of *y*_*i*_ is given by (46, 59, 60).


(1) $${\text{f}}_{\text{i}}({\text{y}}_{\text{ij/}b_{\text{i}},}\beta ,\phi )\;=\text{ exp }\{\frac{{\text{y}}_{\text{ij}}(\theta_{\text{ij}})-\psi (\theta_{\text{ij}})}{\phi }\;+\text{ c }({\text{y}}_{\text{ij}},\phi )\}$$

Here θ_*ij*_ isthe linear predictor ($\theta_{ij}=\;x_{ij}^{'}\beta \;+\;z_{ij}^{'}b_{i}),\;\psi \;(\theta_{ij})$ is the link function, ϕ is the dispersion parameter and *c* (*y*_*ij*_, ϕ) normalizing constant.

The function *g* (μ_*ij*_) is the inverse of the link function ψ (θ_*ij*_). The relationship between g (μ_*ij*_) and *f*_*i*_(*y*_*ij*/_**b**__*i*__, β, ϕ) is given by the following equation:


(2) $$\begin{array}{l} g\;\left(\mu_{ij}\right)\;=\;\int f_{i}\left(y_{ij/{\text{b}}_{i},}\;\beta ,\;\phi \right)\;dy_{ij} \end{array}$$

By using Laplace approximation, Equation 2 approximates to the function;


(3) $$\begin{array}{l} g\;\left(\mu_{ij}\right)\;=\;g\;\left[\epsilon \left(y_{it}|b_{i}\right)\right]\;=\;x_{ij}^{'}\beta \;+\;z_{ij}^{'}b_{i} \end{array}$$

The function *g*(.) is a known link function that belongs to the GLMM framework that is used to map the expected values of the response variable to the linear predictor, *x*_*ij*_ is the *i*^*th*^ row of the matrix for the fixed effects, *z*_*ij*_ is the *i*^*th*^ row of the matrix for the random effects associated with *b*_*i*_, β is the parameter vector of unknown fixed effects and ψ is scall parameter or cumulant generating function.

Under this GLMMs settings, the logit function is commonly chosen as the link function *g*(μ_*ij*_) is defined as


(4) $$\begin{array}{l} g\;\left(\mu_{ij}\right)\;=\text{ logit }\left(\mu_{ij}\right)\;=\text{ log }\left(\frac{\mu_{ij}}{1-\mu_{ij}}\right)\;=\;\eta_{ij}=\;x_{ij}^{'}\beta \;+\;z_{ij}^{'}b_{i} \end{array}$$

Here the conditional expectation equals to the conditional probability of a response given the random effects and covariance values, i.e., μ_*ij*_ = ϵ(*y*_*ij*_*|b*_*i*_, *x*_*ij*_) = *P* (*y*_*ij*_|*b*_*i*_, *x*_*ij*_).

This model can also write as


(5) $$\begin{array}{l} \text{P }\left(y_{ij}|b_{i},x_{ij},\;z_{ij}\right)\;=\;{\text{g}}^{-1}\left(\eta_{iz}\right)\;=\;{\text{g}}^{-1}\left(x_{it}^{'}\beta \;+\;z_{it}^{'}b_{i}\right) \end{array}$$

Where the inverse link functions $g^{-1}(\eta_{ij})$ is the logistic cumulative distribution function (CDF), which is used to quantify the binary response, namely:


(6) $$\begin{array}{l} g^{-1}\left(\eta_{ij}\right)\;=\;{\left[1+\exp\left(\;\eta_{ij}\right)\right]}^{-1} \end{array}$$

In GLMMs, the logistic distribution can facilitate the process of estimating the distribution's parameters by maximum likelihood estimation or other techniques and has the advantage of making a straightforward parameter estimation (61).

#### 2.3.3 Estimation

Likelihood-based approaches rely on the likelihood function to estimate the parameters in GLMMs. With this model, the joint distribution of both the vectors of response and the vectors of random effects are fully specified and we might use the same methods to estimate these models (62). Given the above model specification for the GLMMs based on the assumption that the binary responses *y*_*ij*_ (conditioned on the random effects *b*_*i*_) are conditionally independent, the joint probability of the response vector *y*_*i*_ and the random effect vector *b*_*i*_ for f (*b*_*i*_) distribution of the *i*th random effect can be explained as follows:


(7) $$\begin{array}{l} f\left(y_{i},b_{i}\right)=f\left(y_{ij}|b_{i}\right)f\left(b_{i}\right)=f\left(y_{i1}|b_{i}\right)f\left(y_{i2}|b_{i}\right)\ldots .f\left(y_{in_{i}}|b_{i}\right)f\left(b_{i}\right) \end{array}$$

Then the likelihood function of the parameters β and ${\sigma_{b}}^{2}$ is given by:


(8) $$\begin{array}{c} L\;(\beta ,\;\sigma_{b}^{2})\;=\;\prod_{i=1}^{n}f(y_{i})\;=\;\prod_{i=1}^{n}\int f(y_{i},b_{i})db_{i}\\ =\prod_{i=1}^{n}\int f(y_{i}|b_{i})f(b_{i})db_{i}\\ =\prod_{i=1}^{n}\int \prod_{i=1}^{n}f(y_{it}|b_{i})\;f(b_{i})db_{i} \end{array}$$

Since *y*_*ij*_ is a binary response, has a value of *0* or *1*, The conditional mean of *y*_*ij*_ is related to the linear predictor by a logit link function. Thus, *z*_*ij*_= 1 for all *i*= *1,2… 2,581* and *j*= *1,2…* *n*_*i*_, the linear predictor of Equation 4 was equivalent to:


(9) $$\eta_{ij}=\;x_{ij}^{'}\beta \;+\;z_{ij}^{'}b_{i}=\;x_{ij}^{'}\beta \;+\;b_{i}$$

Thus, Equation 8 can be put in the simplified form as:


(10) $$\begin{array}{c} L\;(\beta ,\;\sigma_{b}^{2})\;=\;\prod_{i=1}^{n}\int \{\exp\left(\beta \sum_{i=1}^{ni}y_{ij}x_{ij}^{'}+y_{i}b_{i}\right)\}\\ \left\{\prod_{j=1}^{ni}\frac{1}{1+exp(x_{ij}^{'}\beta \;+\;b_{i})}\right\}\\ \left\{1/\sqrt{2\pi \sigma_{b}^{2}}exp(-\frac{1}{2\sigma_{b}^{2}})\;b_{i}^{2}db_{i}\right\} \end{array}$$

The values of β and ${\sigma_{b}}^{2}$ that maximize this likelihood function are the ML estimates of β and ${\sigma_{b}}^{2}$. However, from Equation 10, it is not possible to use the entire likelihood function since there are no closed-form solutions. Thus, it is necessary to employ estimates of the probability function to find a solution for this problem. Laplace's approximation approach serves as the foundation for all likelihood-based techniques and the GLMM's parameters are estimated using the glmer function in the lme4 package of R for this likelihood approximation (63, 64).

#### 2.3.4 Laplace's approximation

The Laplace approximation is a quadrature method for estimating integrals of this kind was developed by Laplace and published in 1774,


(11) $$\begin{array}{l} \int_{\text{a}}^{\text{b}}{\text{f}\left(\text{t}\right)\text{e}}^{\lambda \text{g}\left(\text{t}\right)}\text{dt} \end{array}$$

Where both *g(t)* and *f(t)* are continuous smooth functions, *f(t)* is nonzero at *t*_0_, and *g(t)* is a twice-differentiable function on *(a; b)* with a maximum in the interval *(a; b)*. The underlying principle of Laplace's approach is that, for large λ, the integral's bulk will come from the integral's contribution around a certain point, *t*_0_. That resulting integral may be proven to represent the kernel of a normal distribution, which can then be integrated, using second-order Taylor series expansions for *g(t)* and *f(t)*. The integrand in the function is comparable to the likelihood of a GLMMs, which contains exponential functions from the exponential family of probability distributions, as can be seen by examining the form above (63, 64).

#### 2.3.5 Akaike's information criterion

Akaike's information criterion (AIC), is a popular model selection criterion based on likelihood, with the optimal model being the one that minimizes AIC. It frequently works in tandem with the Deviance Information Criterion (DIC) and the Bayesian Information Criterion (BIC) (49). For data set *D* = *{(y*_*i*_,$x_{ij}^{'}$*)}*, where *y*_*i*_ is the outcome vector and $x_{ij}^{'}$ is a set of fixed effects and for maximum likelihood estimator $\hat{\beta }$ under computing model.

For *p* dimension of β, AIC can be formulated as:


(12) $$\begin{array}{l} AIC\;=\;-2L\left(\hat{\beta },D\right)\;+\;2p \end{array}$$

#### 2.3.6 The likelihood ratio test for variance components in GLMMs

GLMM's are used to describe responses from exponential families with a combination of fixed and random effects, and variance components in GLMMs come from random effects (65). This is equivalent to testing that the variance component equals zero and the hypothesis of interest is:


$$\begin{array}{l} H_{O}\;:\;{\sigma_{b}}^{2}\;=\;0\;Vs\;H_{1}:{\sigma_{b}}^{2}\;>\;0 \end{array}$$

For the maximized log-likelihood under the null hypothesis *l*_1_ and the variance component estimated *l*_o_, the test statistics for variance components of the likelihood ratio test are given by:


(13) $$\begin{array}{l} G^{2}=2\left(l_{1}-l_{0}\right) \end{array}$$

Here *G*^2^ follows chi-square distribution with 1 degree of freedom.

## 3 Results

### 3.1 Explanatory data analysis

Exploratory analysis of clustered data intends to identify characteristics of random variation that distinguish individual children as well as patterns of systematic variation across groups of children. Among the main illness categories that were recorded (see Table 2), cough, fever, and diarrheal were found to be the most frequent types of children's illnesses, with percentages of 25.67, 18.52, and 14.08, respectively. Moreover, fast birthing, no stool, difficulty in birth, and swelling occurred at all lower rates under 1 year of age. A total of 2,322 episodes of any illness were noted among the children who were considered in the PMA 2019 survey.

**Table 2:** Distribution of the broad categories of illness among children, PMA-ET 2019 survey.

**Broad illness category**	**Total number of episodes**	**Percentage of episode**	**Mean**	**Episodes/child SD**
Any illness	2,322		1.148	0.036
Cold/cough	596	25.67	0.43	0.136
Fever	430	18.52	0.322	0.109
Diarrhea	327	14.08	0.244	0.082
Vomiting	195	8.4	0.139	0.043
Difficulties feeding/unable to suck	178	7.67	0.131	0.043
Skin rash/skin lesion	170	7.32	0.122	0.038
Red eye/passage of pus from eyes	153	6.57	0.121	0.045
Sore throat/tonsillitis	68	2.93	0.046	0.013
Fast birthing	42	1.81	0.033	0.012
No stool	40	1.72	0.032	0.012
Unconscious	32	1.38	0.005	0.02
Difficulty in birth	31	1.34	0.02	0.005
Reduced alertness (lethargy)	29	1.25	0.025	0.01
Convulsion	11	0.47	0.009	0.004
Abdominal/body swelling	9	0.39	0.007	0.003
Other	11	0.47	0.008	0.003

**Figure 2:**
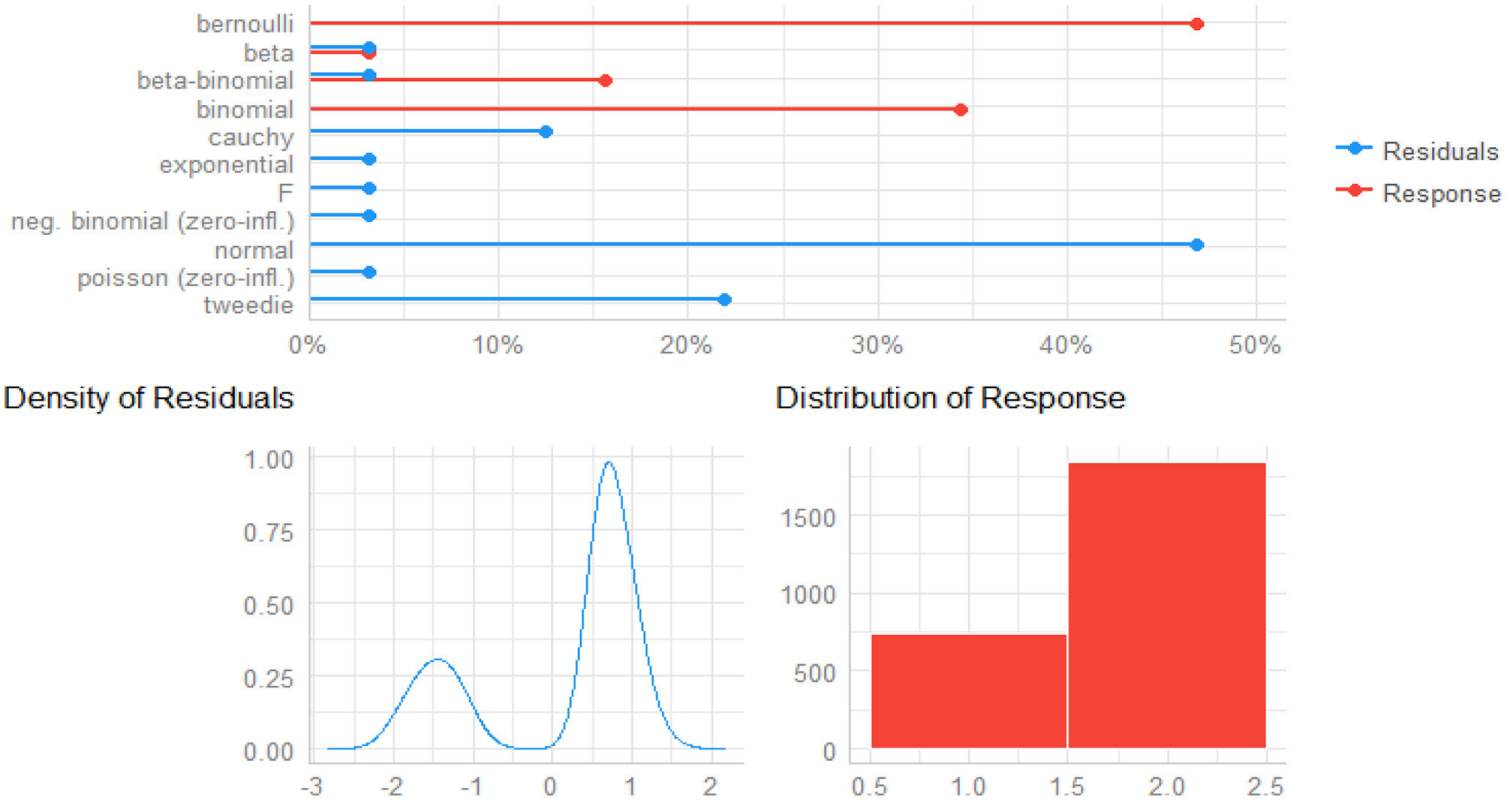
Predicted distribution of residuals and response for child comorbidity study.

The density of residuals and distribution of responses give insight into how the responses and predictors are related to one another (66, 67). As shown in Figure 1, the bottom left of it depicts the density of residuals (see left plot of Figure 2), in which the residuals are obviously bimodal (not normal), and the bottom right side of the plot is the distribution of responses (see right plot of Figure 2). With these distributions, non-normally distributed responses are possible accommodated, including non-linear links between the mean of the child morbidity and the predictors, as well as some form of correlation in the data. Thus, GLMMs with logit link functions are an ideal method of detecting child morbidity for the given datasets.

A Pearson's chi-square test of a bivariate analysis has been carried out to look at the relationship between a few chosen variables (68). The following table (see Table 3) represents the contingency table analysis of the morbidity status of children, along with Pearson's chi-square value to determine if a particular regression coefficient is significant. Mother's age is the only variable that is not significantly (*P*-value = 0.632) related to child morbidity among all the factors that were taken into consideration at the 5% significance level. Furthermore, morbidity is predominant among children whose mothers use charcoal for fuel (37.16%), never attended education (30.20%), live in rural areas (47.77%), and have lower quartiles of wealth (32.70%).

**Table 3:** Characteristics of the study participants by morbidity and their mother's sociodemographic status, PMA-ET 2019 survey (*n* = 2,581).

**Background characteristics**	**Weighted frequency *n* (%)**	**Morbidity status**		**Pearson's *X*^2^ value/*p*-value**
		**No**, ***n*** **(%)**	**Yes**, ***n*** **(%)**	
**Cooking fuel**				
Electricity	435 (16.85)	203 (7.87)	232 (8.99)	chi^2^ (3) = 104.06, Pr < 0.001
Kerosene	10 (0.39)	1 (0.04)	9 (0.35)	
Charcoal	1,764 (68.35)	408 (15.81)	1,356 (52.544)	
Wood	372 (14.41)	130 (5.04)	242 (9.38)	
**Mothers' marital status**				
Married or with partner	442 (17.13)	155 (6.01)	287 (11.1)	chi^2^ (2) = 10.36, Pr = 0.006
Widowed or divorced	810 (31.38)	217 (8.41)	593 (22.9)	
Never married	1,329 (5,149)	370 (14.34)	959 (37.16)	
**Mothers' age**				
15–24	877 (33.98)	264 (10.23)	613 (23.75)	chi^2^ (2) = 1.19, Pr = 0.632
25–34	1,312 (50.03)	369 (14.30)	943 (36.54)	
35+	392 (1,519)	109 (4.22)	283 (10.96)	
**Mothers' education**				
Never attend	986 (38.20)	205 (7.94)	781 (30.20)	chi^2^ (3) = 83.80, Pr < 0.001
Primary	924 (35.8)	258 (10)	666 (25.8)	
Secondary	393 (15.23)	158 (6.12)	235 (9.10)	
Higher or TVET	278 (10.78)	121 (4.69)	157 (6.08)	
**Residence**				
Urban	1,001 (38.78)	395 (15.30)	606 (23.48)	chi^2^ (1) = 90.22, Pr < 0.001
Rural	1,580 (61.22)	347 (13.44)	1,233 (47.77)	
**Wealth quartiles**				
Lower quartile	842 (32.62)	148 (5.73)	844 (32.70)	chi^2^ (2) = 101.79, Pr < 0.001
Middel quartile	400 (15.50)	99 (3.84)	694 (26.89)	
Higher Quartile	1,339 (51.88)	495 (19.18)	301 (11.66)	
**Parity**				
0	518 (20.17)	192 (7.44)	326 (12.63)	chi^2^ (3) = 49.08, Pr < 0.001
1–2	1,031 (39.95)	326 (12.63)	705 (27.31)	
3–4	566 (21.93)	132 (5.11)	434 (16.82)	
5+	466 (18.06)	92 (3.56)	374 (14.49)	
**Sanitation classification**				
Improved, not shared facility	119 (4.61)	52 (2.01)	67 (2.60)	chi^2^ (3) = 70.64, Pr = 0.006
Shared facility	416 (116.12)	180 (6.97)	236 (9.14)	
Non-improved facility	1,170 (45.33)	311 (12,.05)	859 (33.28)	
Open defecation	876 (33.94)	199 (7.71)	876 (33.94)	
**Electricity availability**				
No	1,386 (53.70)	310 (12.01)	1,076 (41.69)	chi^2^ (1) = 59.53, Pr = 0.012
Yes	1,195 (46.30)	432 (16.74)	763 (29.56)	
**Region**				
Addis Ababa			258 (10)	
Afar			222 (8.60)	
Amhara			445 (17.24)	
Oromia			638 (24.72)	
SNNP			587 (22.74)	
Tigray			431 (16.70)	

### 3.2 Generalized linear mixed model analysis

#### 3.2.1 Type-III tests of fixed effects

In GLMMs, Type-III tests are applied to evaluate each term's significance while taking into consideration the effect of every other term (69). Table 4 of the Type III analysis of the likelihood ratio test of all the fixed effects (except sanitation class) significantly affects child morbidity.

**Table 4:** Type III tests of fixed effects from GLMMs of child morbidity, PMA-ET 2019 survey (*n* = 2,581).

**Fixed effects**	**DF**	***F*-values**	**Pr (>*F*)**
Cooking fuel	3	18.8098	0.0005^***^
Wealth	2	19.3282	0.0003 ^***^
Sanitation class	3	0.6979	0.812
Residence	1	4.8428	0.0044 ^**^
Mother education	3	6.9747	0.0016^**^
Marital status	2	4.4209	0.0119 ^*^
Mother's age	2	1.9634	0.018 ^*^
Parity	3	2.7513	0.044^*^
Electricity availability	1	6.0684	0.014 ^*^
Mother education: mother's age	6	1.4051	0.209

^***^p < 0.001, ^**^p < 0.01, ^*^p < 0.05, .*p* < 0.1, *p*≥ 0.1.

The following table (see Table 5) presents the estimates, odds ratio, significance level, and confidence intervals of the estimates of the fixed effects based on the likelihood ratio chi-square test result using the glmer function of the lme4 package in R (70). The estimates tell us the amount of increase in the predicted log odds of comorbidity equals one, which would be predicted by a one-unit increase (going from one category to another category) in the predictor, holding other predictors constant. Based on the results, wealth status significantly affects the child morbidity status, and it is observed that children from middle quartiles (OR = 0.47, *P* = 0.002; 95% CI: −0.766, −0.167) and higher quartiles (OR = 0.62, *P* = 0.001; 95% CI: −1.05, −0.415) are less likely to suffer illness than children from lower quartiles. Our study also demonstrated that children from a mother with primary, secondary, and higher education are 41%, 52%, and 51% respectively, less likely to be ill than mothers who never attended school.

**Table 5:** Estimates of fixed effects from GLMMs for children's comorbidity, PMA-ET 2019 survey (*n* = 2,581).

**Covariates**	**Coef**.	**SE**	***Z***	***P* > |*Z*|**	**OR**	**95% CI (Coef.)**
(Intercept)	0.79	0.33	2.4	0.016 ^*^	2.21	(0.146, 1.44)
**Cooking fuel (ref**.= **electricity)**						
Kerosene	1.62	1.11	1.46	0.144	5.02	(−0.556, 3.78)
Charcoal	0.14	0.20	0.68	0.499	1.14	(−0.258, 0.528)
Wood	0.40	0.16	2.5	0.013 ^*^	1.48	(0.081, 0.712)
**Wealth (ref** = **lower quartile)**						
Middle quartile	−0.47	0.15	−3.1	0.002 ^**^	0.62	(−0.766, −0.167)
Higher quartile	−0.74	0.16	−4.5	0.001 ^***^	0.47	(−1.05, −0.415)
**Sanitation classification (ref**. = **improved, not shared facility)**						
Shared facility	−0.11	0.22	−0.53	0.603	0.89	(−0.548, 0.318)
Non-improved facility	0.06	0.15	0.40	0.693	1.06	(−0.234, 0.351)
Open defecation	−0.03	0.19	−0.14	0.891	0.97	(−0.380, 0.338)
Residence (rural)	0.51	0.18	2.8	0.004 ^**^	1.66	(0.158, 0.858)
**Mother education (ref** = **never attended)**						
Primary education	−0.52	0.22	2.9	0.001^**^	0.59	(−0.946, −0.085)
Secondary education	−0.71	0.25	−2.4	0.018 ^*^	0.48	(−1.21, −0.218)
Higher education	−0.69	0.32	−2.2	0.033^*^	0.49	(−1.34, −0.055)
**Marital (ref**. = **married/partner)**						
Widowed or divorced	0.38	0.14	2.8	0.004 ^**^	1.46	(0.120, 0.648)
Never married	0.32	0.13	2.6	0.010 ^*^	1.37	(0.073, 0.559)
**Mother's age (ref**.= **15–24)**						
25–34	−0.27	0.23	−1.2	0.243	0.76	(−0.719, 0.183)
35+	−0.72	0.27	−2.5	0.010 ^*^	0.49	(−1.26, −0.169)
**Parity (Ref**. = **0)**						
1–2	0.10	0.13	0.80	0.426	1.10	(−0.146, 0.344)
3–4	0.39	0.17	2.3	0.023 ^*^	1.48	(0.055, 0.733)
5+	0.53	0.24	2.5	0.013 ^*^	1.70	(0.111, 0.954)
Electricity availability (NO)	0.40	0.17	2.5	0.014 ^*^	1.49	(0.079, 0.718)

^***^p < 0.001, ^**^p < 0.01, ^*^p < 0.05, .*p* < 0.1, *p*≥ 0.1.

OR, odds ratio; CI, confidence interval; SE, standard error; SD, standard deviation and the model is fitted by maximum likelihood estimation.

Similarly, children who lived in rural areas (OR = 1.66, *P* = 0.004; 95% CI: 0.158, 0.858) are 1.66 times more likely to get affected by morbidity than children who lived in urban areas, and using wood as a fuel is 1.14 times more likely than using electricity to get child morbidity. Likewise, the absence of electricity (OR = 1.49; *P* = 0.014; 95% CI: 0.079, 0.718) is more likely for children's illness as compared to children who can access electricity. This study's findings also suggest that a woman with a parity of 3–4 and 5+, never married, and divorced or widowed mothers' marriage statuses are more likely to have comorbidity than their counterparts.

#### 3.2.2 Interaction effects

The interaction between a mother's education (never attending primary education, secondary education, or higher education) and a mother's age (age between 15 and 24, age between 25 and 34, and age above 35) is presented in Table 6. As the result indicated, children from mothers above 35 years of age are less likely to be ill compared to children whose mother's age is < 34 for secondary and higher mother education groups (OR = 2.3, OR = 1.67, *P*-value = 0.022, *P*-value = 0.015, respectively).

**Table 6:** Estimates of the two-way interaction effects and the variance parameter of the random effect models from GLMMs for child morbidity, PMA-ET 2019 survey (*n* = 2,581).

**Covariates**	**Coef**.	**SE**	***Z***	***P* > |*Z*|**	**OR**	**95% CI of Coef**.
**Education and age (ref** = **never attended: age between 15–24)**						
Primary education: age between 25–34	0.39	0.28	1.5	0.149	1.47	(−0.142, 0.923)
Secondary education: age above 25–34	0.24	0.32	0.76	0.449	1.27	(−0.379, 0.854)
Higher education: age between 25–34	0.03	0.36	0.08	0.935	1.03	(−0.692, 0.751)
Primary education: age above 35+	0.24	0.35	0.67	0.501	1.27	(−0.453, 0.926)
Secondary education: age above 35+	1.26	0.55	2.3	0.022 ^*^	3.53	(0.176, 2.34)
Higher education: age above 35+	0.43	0.65	1.67	0.015^*^	1.65	(0.831, 1.69)
**Random effects**		**Variance**	**SD**			
Region		5.318e−02	0.231			
Child_ID		4.598e−07	0.006			
Residual		0.123	1.045			

^***^p < 0.001, ^**^p < 0.01, ^*^p < 0.05, .*p* < 0.1, *p*≥ 0.1.

OR, odds ratio; CI, confidence interval; SE, standard error; SD, standard deviation; Coef., coefficients.

#### 3.2.3 Model comparison and diagnosis

Comparing the models is an important step in the modeling process to see which ones best fit the data (71, 72). Akaike's information criterion (AIC) is a widely used model selection criteria based on the maximum likelihood estimator (49). Results of the AIC, log-likelihood ratio test (LRT), BIC and other useful information on the fit of the model are presented in Table 7. Accordingly, the model with two random intercepts (the random intercept of region and Child's id) has a lower AIC (AIC = 2,929.9) and is statistically significant (*P* < 0.001) in comparison to one random intercept model (AIC = 2,942.6). It is also supported in the loglikelihood ratio test (LRT) with a significance *P*-value (*P* < 0.001). This suggests that two random intercept models from GLMMs permit data correlation and provide more effective overall performance compared to one random intercept model.

**Table 7:** The Likelihood-Ratio-Test (LRT) and Akaike information criteria for random intercept models comparison from GLMMS of child morbidity (*n* = 2,581).

**Models**	**Akaike information criteria for model comparison**					**Likelihood-ratio-test (LRT) for model comparison (ML-estimator)**		
	**AIC**	**BIC**	**logLik**	**Deviance**	**Pr (**>**Chisq)**	**df**	**Chi** ^2^	**Pr (**>**Chisq)**
ONE RIM	2,942.6	3,106.6	−1,443.3	2,886.6		28		
TWO RIM	2,929.9	3,099.7	−1,435.9	2,871.9	0.001249 ^***^	29	14.72	< 0.001^***^

^***^p < 0.001, ^**^p < 0.01, ^*^p < 0.05, .*p* < 0.1, *p*≥ 0.1.

ONE RIM, one random intercept model; TWO RIM, Two random intercept model.

**Figure 3:**
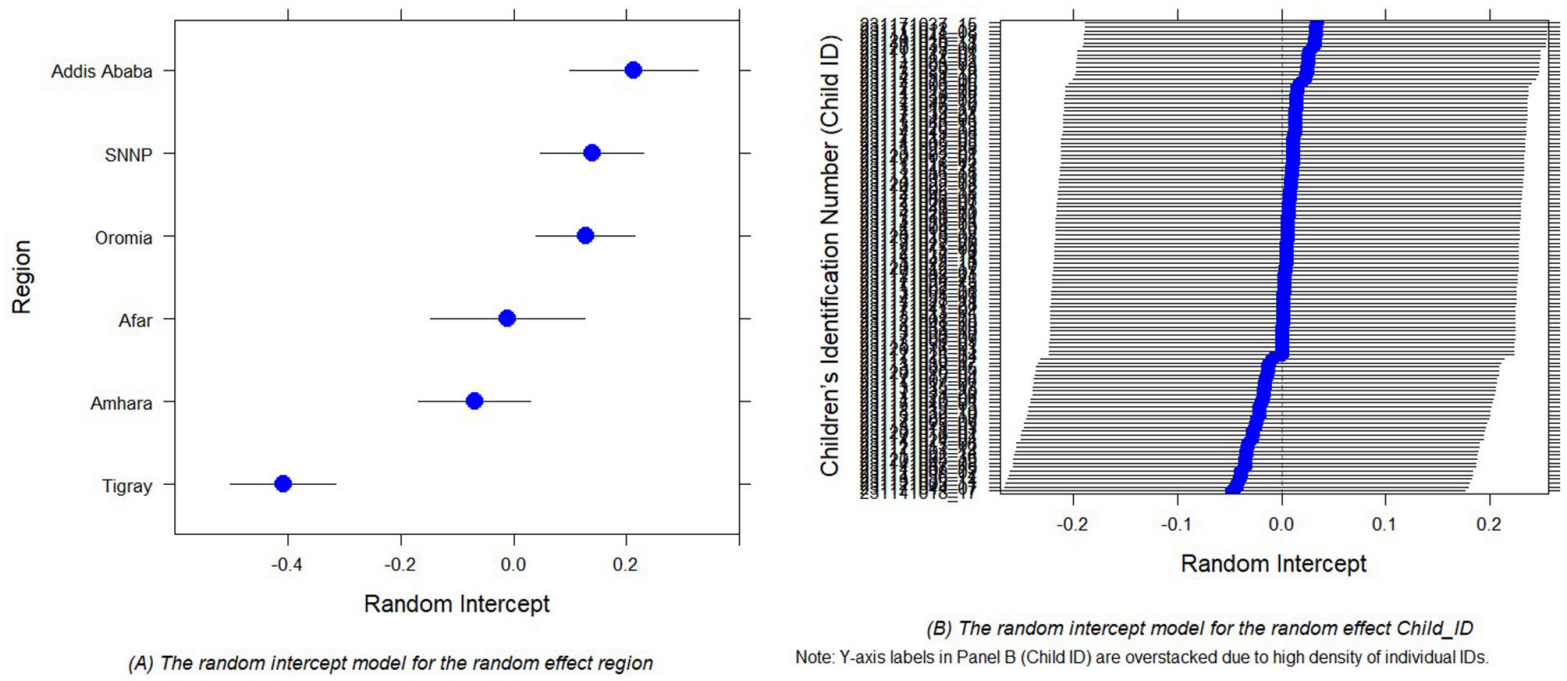
Random intercept plots for the random effect region **(A)** and random effect Child_ID **(B)** of the child comorbidity study.

In GLMMs, random intercept plots are employed to illustrate the distribution of random effects (51, 77). Figures 3A, B displays the diagnostic plots for random intercepts corresponding to two random effects, providing a visual representation of the variability in child morbidity. These plots inform us about the existence of variability at the cluster level for child morbidity. The estimated variance in the intercept, specific to both region and children, is found to be very close to zero. Hence, the inclusion of random effects is a prudent modeling decision, given the considerable variation observed in estimations of both regional and children-specific effects.

#### 3.2.4 Residuals diagnosis in GLMMs

Residuals in GLMMs have a coarse structure due to random effects and grouping of data. As a result, these models should not use techniques like QQ plots or Shapiro–Wilk tests to verify residual normality as standard linear models (73–75). Therefore, we use the “Diagnostics for HierArchical Regression models (DHARMa)' package to create readily interpretable scaled (quantile) residuals for fitted GLMMs (50) and binned residual plots in dividing the data into bins based on fitted value (53).

**Figure 4:**
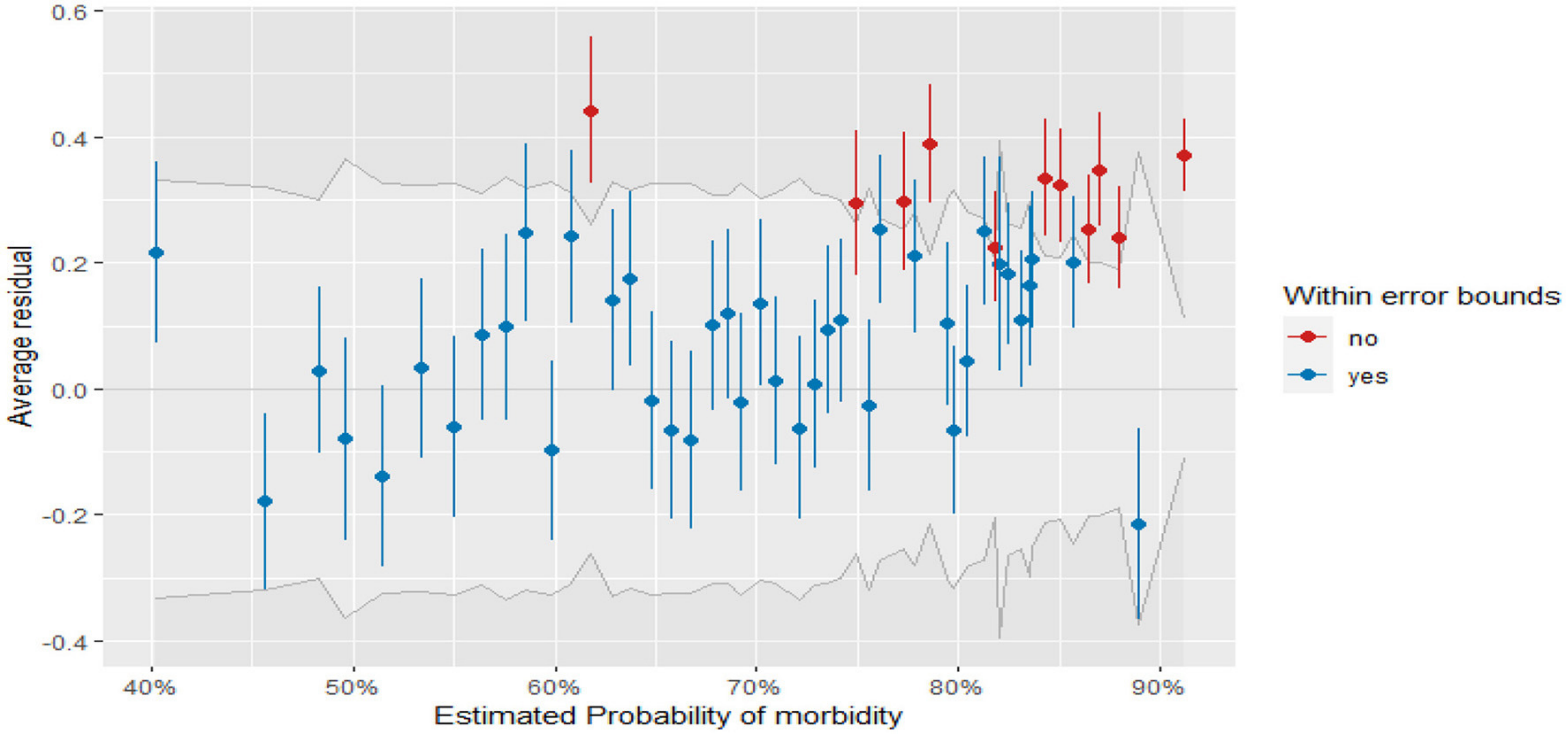
Binned residual plot for the children's comorbidity study.

Figure 4 displays the plots of residuals vs. fitted values for fitted GLMMs (binned residuals). From the plot, most of the residuals fall within the error bound (indicated in blue points), and fewer residuals are outside of the error boundaries (indicated in red points). Thus, most of the binned residual fell within the 95% confidence interval of error bounds, which indicates that the model is a good fit for the data.

**Figure 5:**
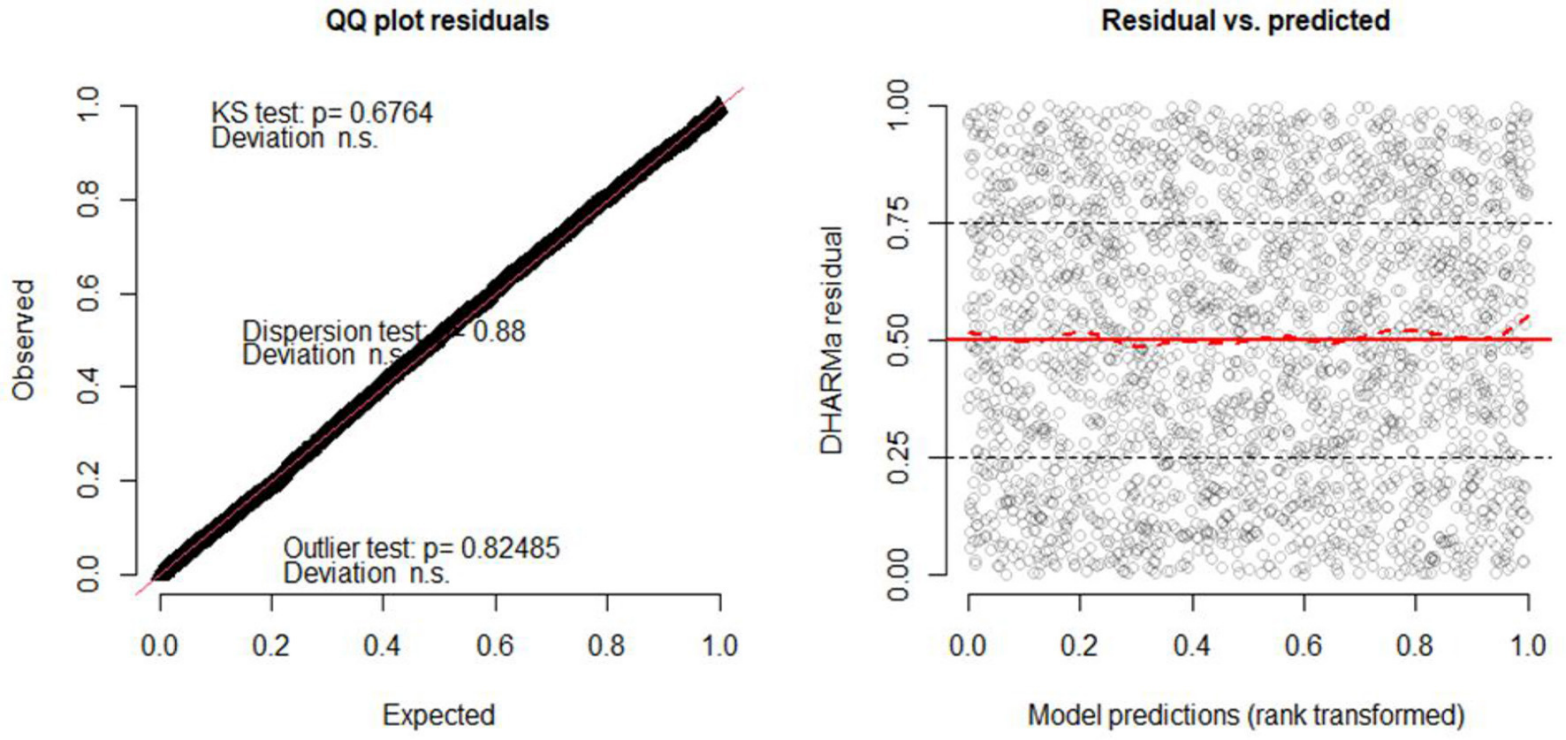
Quartile residuals for children comorbidity study in GLMMs.

Furthermore, in the DHARMa package in R, the QQ plot compares the observed residual to the expected under the assumptions of normality, and the points in the QQ plot fall along a straight line for normally distributed residuals (50, 53). The plot also displays the Kolmogorov-Smirnov test (KS test), dispersion test, and outlier test (76). From Figure 4, the points on the QQ plot fall along a straight line which indicates that the model can account for the variation in child morbidity and the model is not systematically overestimating or underestimating child morbidity (see the left of Figure 5). Moreover, the insignificant values of the KS test, dispersion test, and outlier test (*P* = 0.6764, *P* = 0.88, *P* = 0.82485, respectively) suggest that the residuals of the model are normally distributed, homoscedasticity variance, and no influential observations in the data. Similarly, the right of Figure 5 depicts a plot of the residual against the predicted values. The red solid line at *y* = 0.5 represents the median of the residual, while a dashed red line represents the theoretical median of the residual under the assumption of uniform distribution (78). Therefore, the two lines are close together at *y* = 0.5 indicating that the residuals are uniformly distributed.

## 4 Discussion and conclusion

### 4.1 Discussion

We tried to check the presence of variability in child morbidity and determine major predictive factors for child morbidity using the GLMMs. We used PMA datasets in STATA-17 and the 4.3.0 version of R for our data analysis. Based on AIC and the likelihood ratio test values, a two-random intercept model was found to be more favorable in illustrating the presence of child morbidity variability between children and within regions. From our study using GLMMs, based on the likelihood chi-square and Type III test, we found that the factors that significantly affect the children's comorbidity were cooking fuel, wealth quartiles, mothers' marital status, mother age, parity, residence mother's education status, and availability of electric city. However, sanitation classification is not influential for the presence of children comorbidity in Ethiopia.

Children from divorced and never-married families are at high risk of suffering illness and experiencing more health problems than children from two-partner families. Like studies carried out (29–31), our result suggests that a lack of a stable family structure and the absence of one of her or his family members contribute to the negative effects on children's health. Similarly, our findings demonstrated that children with high parity had a higher risk of morbidity than children with low parity, based on PMA-ET datasets. The study found that increased parity is associated with higher odds of child morbidity, and our result is in accordance with (24, 26) that higher child morbidity is associated with high parity.

Furthermore, the results showed that children who live in rural locations and lack electricity are more likely than their counterparts to experience morbidity difficulty. It demonstrates that living in rural areas and not having access to electricity are positively connected with child morbidity and this result is in accordance with (11, 15, 34). Moreover, the household wealth index has a negative correlation with morbidity in children and it is a significant socioeconomic determinant influencing children's health in Ethiopia. The lower quartile families had bad nutrition, limited education, poor cleanliness, and poor hygiene. This suggests that compared to children from middle and high quartiles, children from lower households are more likely to experience children's illness. The findings align with those reported by Chalasani and Rutstein (20), Hong et al. (23), and Takele et al. (27), indicating that an increase in household income is associated with a reduction in the incidence of illness among children.

The results we found also showed a negative correlation between childhood morbidity and the age of the mother. This suggests that children whose mothers were younger than 24 have a higher rate of illness. Our findings support the findings of Hviid et al. (79), who noticed that children of mothers 35 years of age and older had lower rates of child morbidity than children of younger mothers. However, our results also contradict those of Nourkami-Tutdibi et al. (80), who found that children of mothers 35 years of age and older had higher rates of child morbidity than children of younger mothers. Another significant risk factor for children's comorbidity is the mother's academic achievement. The risk of morbidity is higher in children whose mothers have not received any education compared to children whose mothers have completed at least primary education. It implies that educated mothers are also more likely to have an income and better access to child health care and have access to information about the health, eating habits, and development of their children, which can enhance the health of their children. These results confirm the results obtained from previous studies (21, 22, 25).

This study has several limitations that should be considered when interpreting the findings. The sampling design of the PMA-ET survey may have introduced selection bias, potentially leading to an overrepresentation of mothers with better access to health services, higher education, and urban residence. This could have affected both internal and external validity, likely underestimating child morbidity among disadvantaged and hard-to-reach populations. Furthermore, child morbidity was assessed through maternal self-reports without objective clinical verification, introducing the possibility of information bias. Symptoms may have been underreported due to recall or social desirability bias, or alternatively overreported by more health-conscious mothers, resulting in potential underestimation or overestimation of true morbidity rates. Additionally, some important confounders, such as water quality, sanitation practices, and access to prenatal care, were not directly measured or controlled, raising the risk of residual confounding that could bias associations in either direction.

The cross-sectional nature of the data further limits the ability to establish clear causal relationships, raising concerns about reverse causality. For instance, while poor household conditions might increase child morbidity, it is also plausible that caring for a sick child could lead to economic strain and worsening household circumstances. Moreover, although missing data were minimal, we evaluated their presence through frequency checks and data summaries to ensure the completeness of key variables. No formal sensitivity analysis was conducted, as the missingness was negligible and unlikely to influence the robustness of the findings. Future studies should prioritize longitudinal designs, include objective clinical validation of child health outcomes, and capture a wider range of confounding factors to better clarify causal pathways and improve the generalizability of results.

### 4.2 Conclusion

According to our result, GLMMs are better suited to handle complex data structures like hierarchical data. This model also offers more precise estimates of random effects on this child comorbidity study to capture heterogeneity and look at how it relates to different variables like socioeconomic status, use of health services, and health outcomes. Cooking fuel, wealth quartiles, mothers' marital status, mother age, parity, residence mother's education status, and availability of electric city were significantly associated with children's morbidity. Improving the socio-economic standings of mothers through socio economic and education reduces the prevalence of child morbidity under the age of one.

## Data Availability

The datasets presented in this study can be found in online repositories. The names of the repository/repositories and accession number(s) can be found at: https://www.pmadata.org/data/available-datasets.
